# RG-I Domain Matters to the In Vitro Fermentation Characteristics of Pectic Polysaccharides Recycled from Citrus Canning Processing Water

**DOI:** 10.3390/foods12050943

**Published:** 2023-02-23

**Authors:** Jiaxiong Wu, Sihuan Shen, Qiang Gao, Chengxiao Yu, Huan Cheng, Haibo Pan, Shiguo Chen, Xingqian Ye, Jianle Chen

**Affiliations:** 1 Ningbo Innovation Center, Zhejiang University, Ningbo 315100, China; 2 College of Biosystems Engineering and Food Science, National-Local Joint Engineering Laboratory of Intelligent Food Technology and Equipment, Zhejiang Key Laboratory for Agri-Food Processing, Fuli Institute of Food Science, Zhejiang Engineering Laboratory of Food Technology and Equipment, Zhejiang University, Hangzhou 310058, China; 3 Zhejiang University Zhongyuan Institute, Zhengzhou 450000, China; 4 Innovation Center of Yangtze River Delta, Zhejiang University, Jiaxing 314102, China; 5 Shandong Huihuang Food Co., Ltd., Linyi 276000, China

**Keywords:** rhamnogalacturonan-I, pectic polysaccharides, citrus processing, in vitro fermentation gut microbiota

## Abstract

Canned citrus is a major citrus product that is popular around the world. However, the canning process discharges large amounts of high-chemical oxygen demand wastewater, which contains many functional polysaccharides. Herein, we recovered three different pectic polysaccharides from citrus canning processing water and evaluated their prebiotic potential as well as the relationship between the RG-I domain and fermentation characteristics using an in vitro human fecal batch fermentation model. Structural analysis showed a large difference among the three pectic polysaccharides in the proportion of the rhamnogalacturonan-I (RG-I) domain. Additionally, the fermentation results showed that the RG-I domain was significantly related to pectic polysaccharides’ fermentation characteristics, especially in terms of short-chain fatty acid generation and modulation of gut microbiota. The pectins with a high proportion of the RG-I domain performed better in acetate, propionate, and butyrate production. It was also found that *Bacteroides*, *Phascolarctobacterium*, and *Bifidobacterium* are the main bacteria participating in their degradation. Furthermore, the relative abundance of *Eubacterium_eligens_group* and *Monoglobus* was positively correlated with the proportion of the RG-I domain. This study emphasizes the beneficial effects of pectic polysaccharides recovered from citrus processing and the roles of the RG-I domain in their fermentation characteristics. This study also provides a strategy for food factories to realize green production and value addition.

## 1. Introduction

Citrus fruits, including orange (*Citrus sinensis*), tangerine or mandarin (*Citrus reticulata*), grapefruit (*Citrus vitis*), lemon (*Citrus limonum*), and lime (*Citrus aurantifulia*), are among the most extensively cultivated and consumed fruits around the globe [1,2,3]. Some citrus fruits are consumed directly by people, and the rest are processed into products in different forms. Citrus canning has a dominant position in the citrus processing industry, and the total export of canned citrus in China is 265,800 tons, accounting for over 50% of the total global amount [4,5]. The most important step of citrus canning processing is eliminating segment membranes, and the traditional process is chemical hydrolysis with hydrochloric acid and sodium hydroxide. This process is more practical and economical for industrial manufacture. However, the chemical treatment method generates substantial amounts of processing water, which is rich in pectic polysaccharides and polyphenols [5,6]. The waste-processing effluents are disposed of via different methods, such as by releasing them into rivers directly and discharging them into the city sewage system, thereby leading to many environmental problems [7]. Therefore, processing industries need to efficiently extract or recycle these value-added compounds or phytochemicals.

Pectin, an acidic heteropolysaccharide, is abundant in the cell wall of higher plants and is extensively used in the food industry as a gelatinizer, stabilizer, emulsifier, and fat substitute [8,9,10]. Structurally, pectin comprises the homogalacturonan (HG), rhamnogalacturonan-I (RG-I), and rhamnogalacturonan-II (RG-II) domains [11]. As the “smooth region” of pectin, HG is a linear chain comprising α-1,4-D-galacturonic acid (GalA) with different methoxycarbonyl groups. RG-I is the “hairy region” of pectin and is formed by the backbone of repeating disaccharides of GalA and α-1,2-L-rhamnose (Rha) and the side chains of neutral sugars (such as α-L-arabinose and galactose). RG-II, which is also the “hairy region” of pectin, comprises the GalA chain and various sugars such as xylose, fucose, and apiose. 

Pectin is gradually being considered as a prebiotic candidate because of its indigestibility and microbiota accessibility [12,13]. Pectin has been confirmed to enable improvements in inflammatory bowel disease, obesity, and cancer, probably due to its fermented metabolites [8,14]. Moreover, several studies have pointed out that slowly fermentable and highly polymerized carbohydrates may be more important than quickly fermentable carbohydrates. The reason may be that the lack of fermentable carbohydrates in the distal colon caused by carbohydrates’ fast fermentation will induce gut microbiota to degrade protein and produce harmful metabolites, but slowly fermentable carbohydrates can reach the distal colon and be fermented there [15,16,17]. Many studies have also reported the biological activities of RG-I-type pectic polysaccharides, which may be attributed to the function of neutral sugar chains. These studies have found that arabino-oligosaccharides and galactosan can regulate gut microbiota and be considered as potential prebiotics [18,19]. Therefore, studying the fermentation characteristic of pectins (especially RG-I-type pectic polysaccharides) is important and meaningful. 

The relationship between the structure and function of pectin is attracting research attention, and many studies have focused on pectin obtained using different extraction methods (e.g., ultra high pressure technology and microwave irradiation-associated extraction technology) or different sources (such as citrus, Ganoderma atrum, and seaweed) to explore the influence of its structure on fermentation characteristics [20,21,22,23]. Our previous study found that the structure of pectic polysaccharides from three citrus canning processes was different, which might lead to differences in functions [4]. However, most studies did not consider the diversity of factors and the individual effects of different pectins’ molecular weight (Mw), degree of esterification (DE), or monosaccharide composition on their fermentation characteristics, which still remain unclear. Herein, we extracted three different RG-I-type pectic polysaccharides from citrus canning wastewater. Structurally, the Mw and DE of these three pectins were similar, and the most significant difference among them was found in the proportion of the RG-I domain. Then, we evaluated their fermentation characteristics using an in vitro human fecal batch fermentation model and found that the RG-I domain was crucial for pectin in short-chain fatty acid (SCFA) generation and modulation of gut microbiota. This study aims to explore the beneficial effects of reclaimed RG-I pectic polysaccharides and further clarify the structure–activity relationship.

## 2. Materials and Methods

### 2.1. Materials and Chemicals

Satsuma mandarin (*Citrus unshiu* Marc. *Owari satsuma*) and sweet orange (*Citrus sinensis* (L.) Osbeck) were obtained from the Citrus Research Institute of Zhejiang Province, China. Fucose (Fuc), rhamnose (Rha), arabinose (Ara), galactose (Gal), glucose (Glc), mannose (Man), glucuronic acid (GlcA), galacturonic acid (GalA), xylose (Xyl), SCFA standards, 1-phenyl-3-methyl-5-pyrazolone (PMP), and fructooligosaccharide (FOS) were all purchased from Sigma-Aldrich (Shanghai, China). All other chemicals used in this study were analytically pure or chromatographically pure.

### 2.2. Extraction of RG-I Pectic Polysaccharides from Citrus Segment Membranes

From our previous study published by Shen et al. [4], three different pectic polysaccharides rich in the RG-I domain from segment membranes of satsuma mandarin and sweet orange were extracted using a novel citrus canning processing technology (recycling wastewater for electrolyte beverage production). The monosaccharide composition of the pectic polysaccharides is presented in Table 1. The content of the RG-I domain was calculated using the formula RG-I (%) ≈ 2Rha(mol%) + Ara(mol%) + Gal(mol%). On the basis of the proportion of the RG-I domain, these pectic polysaccharides were named RG-46, RG-56, and RG-67. 

RG-46 was extracted from satsuma mandarin segment membranes using the acid–alkali sequential extraction method. In detail, mandarin segment membranes were first treated with 0.4% citric acid and 0.1% HCl for 40 min at 30 °C. After filtration, the membranes were treated with 0.2% NaOH and 0.1% KOH for 10 min. Then, the alkaline extracting solution was precipitated overnight with 95% ethanol at a volume ratio of 1:1. The sediments were redissolved in ultrapure water and then dialyzed with 500-mesh dialysis bags for 48 h. After vacuum freeze drying, RG-46 was obtained. RG-67 was extracted from sweet orange segment membranes under similar conditions (the only difference was that the sweet orange segment membranes were treated with 0.4% citric acid and 0.1% HCl for 50 min rather than 40 min). RG-56 was extracted by repeating the extraction method of RG-67 three times according to the circulating water system used in factory production.

### 2.3. Monosaccharide Composition Analysis

According to the method proposed by Yan and colleagues [6], monosaccharide composition analysis was carried out. About 1 mL of pectic polysaccharide solution (2–3 mg/mL) or fermented supernatant was mixed with 4 mol/L trifluoroacetic acid (TFA, 1 mL) into an ampoule and then sealed using an alcohol blast burner. The system was hydrolyzed at 110 °C for 8 h. After cooling, samples were dried with methanol and blown with nitrogen to remove redundant TFA. Then, 1mL of distilled water was added to redissolve monosaccharides and mixed with 1 mL of PMP solution (0.5 mol/L) at 70 °C for 30 min. After derivation, the system was leached with chloroform three times to remove excrescent PMP. The supernatant was filtered using 0.22 μm membranes and analyzed via HPLC (Waters, Milford, MA, USA). The HPLC system was equipped with a Zorbax Eclipse XDB-C18 column for monosaccharide analysis. Solvent A was a 0.05 mol/L KH_2_PO_4_ buffer (adjusted pH of 6.80) with 15% (V:V) acetonitrile, and solvent B was the same buffer with 40% (V:V) acetonitrile. The flow rate was 1 mL/min, and the column temperature was 25 ± 5 °C. The elution program was 0–15% B (0–10 min), 15–25% B (10–30 min), and 25–0% B (30–45 min). All detections were carried out at 250 nm.

### 2.4. Molecular Weight Analysis

High-performance size-exclusion chromatography (HPSEC) was used to measure the molecular weight. Pectic polysaccharide samples or supernatants of fermented medium were filtered using 0.22 μm membranes and then detected with a multi-angle laser light scattering and refractive index detector (MALLS-RI) at 40 °C. A Shodex OHpak SB-G column, SB-804 HQ (10 μm, 8.0 × 300 mm, exclusion limit 1 × 10^6^) column, and SB-806 HQ (13 μm, 8.0 × 300 mm, exclusion limit 2 × 10^7^) column were used together for analysis. All samples were eluted with 0.15 mol/L NaCl solution containing 0.02% proclin at the flow rate of 0.5 mL/min for 60 min. The dn/dc value used here was 0.138. All data analyses were conducted using ASTRA software (Version7.1.8, Wyatt Technologies Co., Santa Barbara, CA, USA).

### 2.5. Degree of Esterification Analysis

The degrees of methyl esterification (DM) and acetylation (DA) were determined together via HPLC (Waters, Milford, MA, USA) [24]. In brief, 5 mg of pectic polysaccharide was mixed with 0.5 mL of an aqueous solution containing 10 mmol/L CuSO_4_ and 10 mmol/L isopropanol. After mixing thoroughly, 0.5 mL of NaOH (1 mol/L) was added to the system. Then, the system was incubated at 4 °C for 30 min to initiate saponification and then centrifuged at 8000× *g*/min for 10 min. The supernatant was obtained, and the pH was adjusted to 3. After filtering with 0.22 μm filter membranes, the samples were used for analysis. The internal standard method was used for determination, and thus the response factors of methanol and acetic acid needed to be measured first. A chromatographic mixture of methanol, acetic acid, and isopropanol with a mass ratio of 3:1:1 was prepared and injected into the HPLC system. The HPLC system was equipped with a C18 column (SinoChrom ODS-BP, 5 μm, 250 × 4.6 mm) and refractive index detector (RID). The mobile phase used was 4 mmol H_2_SO_4_, and the flow rate was 0.8 mL/min. The column temperature was 30 °C, and elution time was 30 min. The calculation formulas were as follows:

FR = (M_MeOH_ or M_HAc_ × A_IPA_)/(M_IPA_ × A_MeOH_ or A_HAc_)

DM = (FR × A_MeOH_ × M_IPA_ × 1,760,000)/(M_Sample_ × A_IPA_ × GalA% × 32)

DA = (FR × A_HAC_ × M_IPA_ × 1,760,000)/(M_Sample_ × A_IPA_ × GalA% × 60)

where FR is the response factor, MeOH represents methanol, HAc represents acetic acid, IPA represents isopropanol, Sample represents different pectic polysaccharides, A represents the peak area, M represents mass, and GalA% represents the mass fraction of galacturonic acid in the samples. 

### 2.6. In Vitro Human Fecal Microbiota Fermentation

The fecal microbiota fermentation experiment was carried out according to the method described by Yu et al., with some amendments [25]. An amount of 35 mg of FOS (as a positive control group) and RG-46, RG-56, and RG-67 were added to 6.3 mL of carbon-free medium (10.0 g/L casein peptone, 2.5 g/L yeast extract, 1.5 g/L NaHCO_3_, 1.0 g/L cysteine-HCl, 0.9 g/L NaCl, 0.45 g/L KH_2_PO_4_, 0.45 g/L K_2_PO_4_, 0.09 g/L MgSO4·7H_2_O, 0.09 g/L CaCl_2_, 10.0 mg/L hemin, 10.0 mg/L vitamin B_6_, 5.0 mg/L vitamin B_2_, 5.0 mg/L p-aminobenzoic acid, 2.0 mg/L vitamin B_7_, 2.0 mg/L folic acid, 0.8 mg/L resazurin solution, and 0.1 mg/L vitamin B_12_) and then mixed thoroughly. Fresh human excrement was collected from six healthy donors (3 men and 3 women, 18 < BMI < 24) who did not have any gastrointestinal diseases and had not taken antibiotics in the last three months. The excrement was dissolved in PBS (10%, *w*/*v*) and then filtered with four layers of gauze after homogenization. An amount of 0.7 mL of the fecal slurry was immediately added to 6.3 mL of medium to make sure that the final concentration of polysaccharides was 5 mg/mL. All samples were incubated at 37 °C in an anaerobic chamber and sampled at 0 h, 4 h, 8 h, 12 h, and 24 h during the fermentation. After centrifugation (10,000× *g*, 5 min, 4 °C) and pH measurement, the supernatant and pellet were kept at −80 °C for further analysis. All samples were analyzed in triplicate in the anaerobic chamber.

### 2.7. Measurement of pH, SCFAs, and Total Sugar

The fermentation supernatant of different fermentation periods was taken to determine the pH degree. The supernatant was diluted fourfold and then filtered using hyperfiltration membranes for the determination of SCFAs. The short-chain fatty acid concentrations of all groups were analyzed using an Agilent 6890 N GC-FID, which was equipped with an HP-INNOWAX column. Nitrogen was the carrier gas, and the flow rate was 20 mL/min. The inlet temperature and detector temperature were both 240 °C. The initial oven temperature was 100 °C, which was then heated to 180 °C at a rate of 4 °C/min. The injection volume of each sample was 1 μL, and the running time was 20.5 min. The SCFA concentrations were calculated according to the standard curve formed by the concentration peak area.

The total sugar content was determined using the phenol-sulfuric acid method. In brief, 1 mL of 6% phenol solution and 5 mL of concentrated sulfuric acid were added to 1 mL of fermented supernatant. The system was mixed evenly and heated in a boiling water bath for 10 min, and then the absorbance value was measured at 490 nm.

### 2.8. Gut Microbiota Analysis

The gut microbiota composition was analyzed in samples collected from the 24 h fermentation. The samples were centrifuged at 10,000× *g*/min for 5 min to obtain the fecal pellets. Total bacterial DNA was extracted using a TIANamp Stool DNA Kit. All extracted DNA samples were sent to the analysis company for quality identification and gut microbiota analysis. The V4 region of 16S rDNA was selected for amplification, and the Illumina Miseq platform was used for high-throughput sequencing and bioinformatics analysis. All analyses were based on sequencing reads and operational classification units (OTUs).

### 2.9. Statistical Analysis

All experiments were repeated three times in parallel. The data are expressed as the mean ± standard deviation. Statistical analysis was accomplished using SPSS Statistics 26 software (IBM Corp, Armonk, NY, USA), and the significance of the differences was evaluated using one-way ANOVA (*p* < 0.05). Origin 8.0 software (OriginLab Corp., Northampton, MA, USA) and GraphPad Prism 8 software (Graphpad Corp., Boston, MA, USA) were used for mapping.

## 3. Results and Discussion

### 3.1. Analysis of Monosaccharide Composition, Molecular Weight, and Degree of Esterification

The monosaccharide composition of the three pectic polysaccharides with different proportions of the RG-I domain (RG-46, 56, and 67) is shown in Table 1. Large differences existed in the monosaccharide composition among RG-46, RG-56, and RG-67. GalA, Ara, and Gal were the main monosaccharides, accounting for over 30%, 20%, and 10% of the total monosaccharides, respectively. Although the Rha amount was significantly lower than that of the above monosaccharides, it was indispensable to the pectic polysaccharides’ primary structure. The chain backbone of the RG-I domain was composed of repeating GalA and Rha [26]. Apparently, a higher content of Rha meant a longer chain in the RG-I domain. The side chains were composed of neutral sugars (Gal and Ara) intertwined with each other and absorbed into the backbone, forming the RG-I domain [27]. Clearly, the content of Ara in RG-67 was significantly higher than that in RG-46 and RG-56. According to the formulas, the molar percentages of the HG and RG-I domains were calculated. The proportion of the HG domain in RG-46 was significantly higher than that in the other samples, and the proportion of the RG-I domain in RG-67 was the highest.

The molecular weight of the three pectic polysaccharides is listed in Table 1. The molecular weight of three RG-I-type polysaccharides was very close, and the order was RG-67, RG-56, and RG-46 from high to low. This finding may be related to the primary structure and multistage structure of polysaccharides; for example, the manifold and long neutral sugar chains in RG-67 resulted in its relatively large Mw. It was found that the Mw and Mn were positively correlated with the proportion of the RG-I domain. Although the Mw of the three RG-I polysaccharides were close, the difference in the Mn among them was significant, leading to varying polydispersity (Mw/Mn). A larger polydispersity corresponds to a wider sample distribution [28]. Therefore, RG-46 was evenly distributed, while RG-56 and RG-67 were more widely distributed.

The degrees of methyl esterification (DM) and acetylation (DA) were also measured. It was obvious that the DM of these three pectic polysaccharides was small, accounting for only about 10%. The DM was related to the proportion of the RG-I domain, and RG-67 had the highest degree. Meanwhile, the value of DA was extremely low and could not even be detected in RG-56 and RG-67. This indicates that a low esterification degree and low acetylation degree are distinguishing features of pectic polysaccharides extracted via the alkaline process, on account of the saponification reaction [29].

### 3.2. pH Change and SCFA Generation during Fermentation

**Figure 1:**
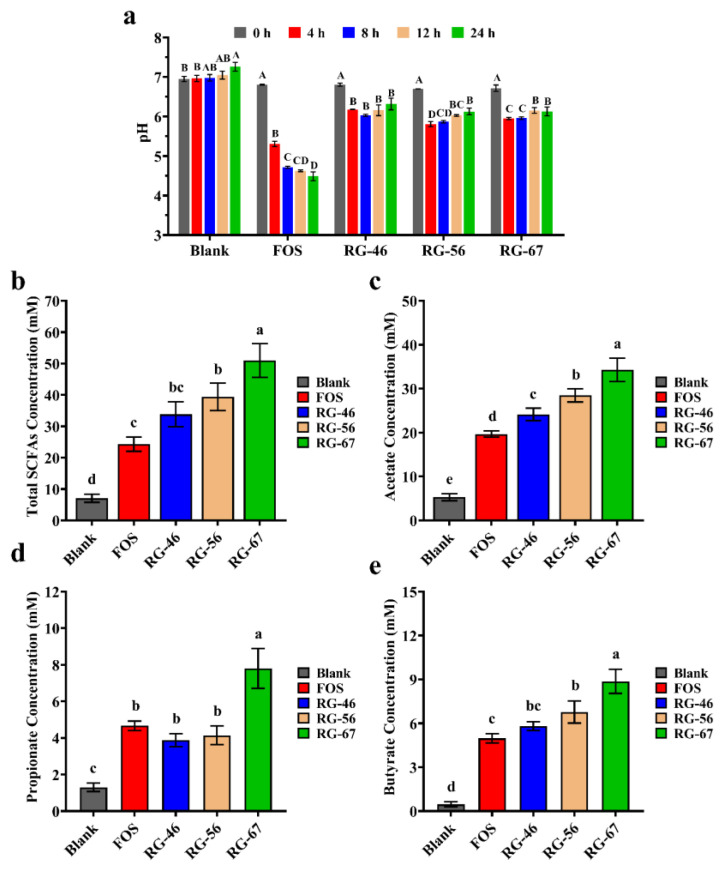
The change in pH and SCFA accumulation during fermentation. (**a**) The change in pH; (**b**) the concentrations of total SCFAs in the fermentation supernatant; (**c**–**e**) the concentrations of acetic acid, propionic acid, and butyric acid. Different uppercase letters indicate significant differences among different fermentation times (*p* < 0.05). Different lowercase letters indicate significant differences among different samples (*p* < 0.05).

The initial pH of each group was within 6.8 to 7.2 (as shown in Figure 1a). The pH of FOS and all pectic polysaccharides decreased during fermentation, especially within 0 to 4 h. The results suggested that the pH of FOS gradually decreased during the whole in vitro fermentation. However, the pH changes of RG-67, RG56, and RG-46 did not reflect those of FOS; they tended to be constant or slightly increase within 12 to 24 h. The final pH decline degree of RG-67 was relatively greater than that of the other samples.

During fermentation, the substrates were degraded by gut microbiota and transformed into a series of complex metabolites. SCFAs, as the main metabolites in carbohydrate metabolism, benefit human gut health by maintaining intestinal homeostasis, improving metabolic syndrome, and exerting anti-inflammation and antitumor activities [30,31,32]. The concentrations of SCFA accumulation during fermentation are shown in Figure 1b–e. Obviously, all substrates could significantly promote the production of SCFAs, and the concentration increased with fermentation. The total SCFA concentration in all samples at 24 h was significantly higher than that in the blank group. Among the four samples, the order of the total SCFA concentrations at 24 h was RG-67, RG-56, RG-46, and FOS from high to low. These results suggested that RG-I-type pectic polysaccharides could produce more SCFAs than the traditional prebiotic FOS, and that the proportion of the RG-I domain is positively correlated with SCFA production. 

Acetate was the dominant SCFA (as shown in Figure 1c), followed by propionate and butyrate (as shown in Figure 1d,e), whereas the contents of isobutyrate, valerate, and isovalerate were very low [33]. Compared to FOS and the other two RG-I pectins, RG-67 performed the best in SCFA production, regardless of whether it was acetate, propionate, or butyrate. The growth trends of acetate and butyrate among the groups were similar to the results of the total SCFAs, revealing that a high RG-I domain proportion could lead to more acetate and butyrate production. However, this correlation between the RG-I domain and SCFAs was not shown in propionate production. These results might be related to the different bacteria involved in the degradation of different RG-I pectic polysaccharides.

### 3.3. Degradation of Pectic Polysaccharides during Fermentation

**Figure 2:**
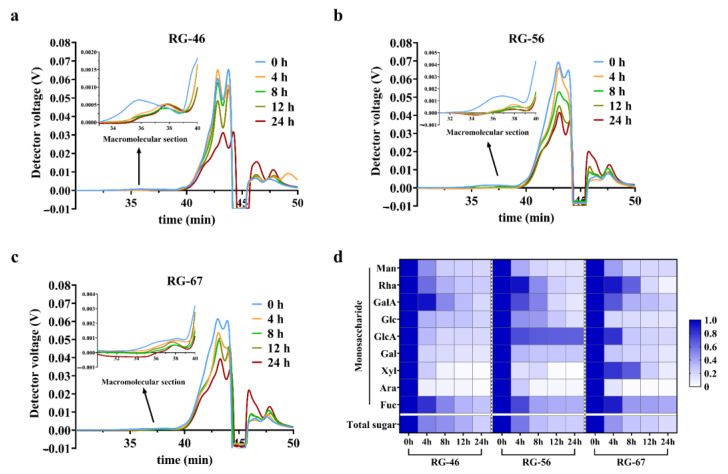
Consumption and degradation of three pectic polysaccharides. (**a**–**c**) The degree of molecular weight reduction of RG-46, RG-56, and RG-67 detected by SEC-MALLS-RI; (**d**) relative ratio of total sugars and monosaccharides remaining during fermentation compared with the contents at 0 h.

The results of the HPSEC in RG-46, RG-56, and RG-67 showed a backward retention time and decreased corresponding peak area with fermentation (Figure 2a–c). This indicates that the molecular weight and molar mass of the pectic polysaccharides gradually decreased during fermentation. During the whole fermentation, 0–4 h was the main period for the degradation of pectic polysaccharides, and nearly half of the substrates were utilized by microbiota. A low Mw fraction at 40–43 min also indicated remarkable degradation. Notably, RG-67 showed a slower degradation speed, although it was almost exhausted before the end of fermentation. Considering its SCFA accumulation, this could be explained by the slow fermentability of RG-67, which was associated with solubility. 

According to the analysis of the monosaccharide composition at different time points, the relative percentage of monosaccharides remaining from 4 h to 24 h was analyzed and compared with the amounts at 0 h. Microbiota manifested a similar preference to monosaccharides among RG-46, RG-56, and RG-67. Specifically, arabinose, xylose, galactose, and mannose in the pectic polysaccharides were more easily utilized, but galacturonic acid, rhamnose, fucose, glucose, and glucuronic acid were relatively more difficult to degrade. We noted that gut microbiota possessed distinct sugar preferences, and neutral sugars were more easily selected than acidic sugars, which is consistent with previous findings [34,35]. The total carbohydrate contents are also shown in the diagram, indicating that the sugar utilization rates differed among groups. It was noted that pectins with a high RG-I domain proportion showed a relatively slow degradation speed. These results were in accordance with the Mw degradation and utilization of monosaccharides, meaning that a high proportion of the RG-I domain prolonged the fermentation and metabolized into more SCFAs by microbiota after fermentation. The concentration and sites of SCFA generation were found to be vital for human gut health, and the lack of carbon sources could promote bacteria to degrade protein and nitrogen metabolism and generate numerous noxious gases to harm gut health [15]. Based on the above results, RG-I pectic polysaccharides (especially RG-67) might benefit humans by prolonging fermentation and promoting the level of fermentable carbohydrates in the distal colon.

### 3.4. Diversity and Composition Analysis of Gut Microbiota

In response to the effect of gut microbiota, all four substrates were degraded to varying degrees and transformed into a series of metabolites. However, these substrates and metabolites modulated the composition of the gut microbiota in turn. The alpha diversity of gut microbiota among the five groups is shown in Table 2. Sobs, Chao, and Ace are the indices of community richness, reflecting the richness of the microbiota composition in the groups. Clearly, RG-46, RG-56, and RG-67 were better than FOS in facilitating microbiota richness. Shannon, Simpson, and Heip are the indices of community diversity. A higher value of the Shannon and Heip indices or a lower value of the Simpson index indicates the samples’ superiority in microbiota diversity. In summary, RG-46, RG-56, and RG-67 all had better performances than FOS in increasing bacteria diversity.

**Figure 3:**
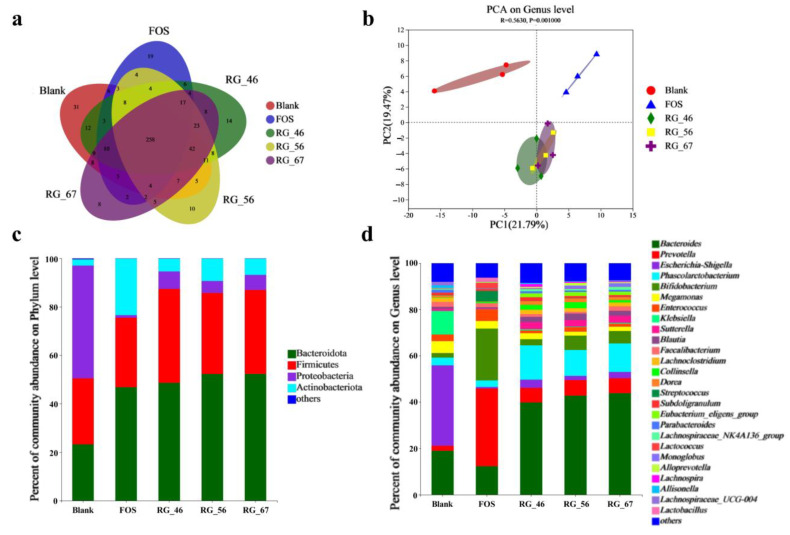

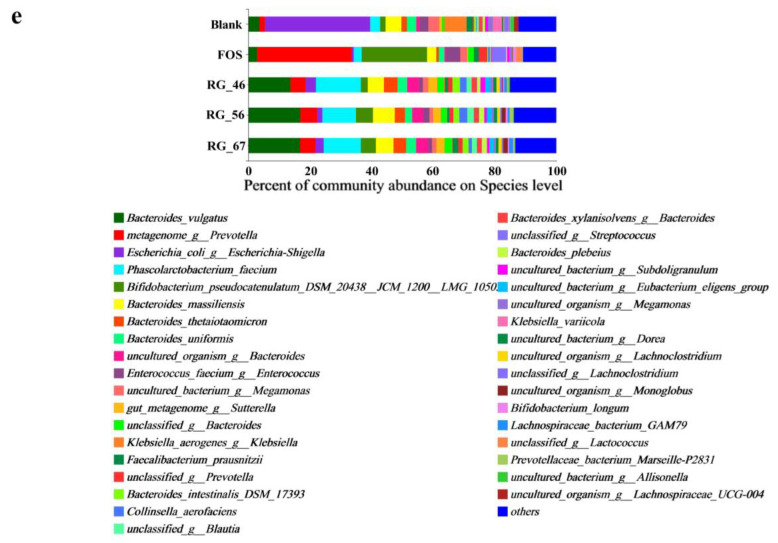
Composition analysis of gut microbiota. (**a**) Venn diagram of OTUs; (**b**) the result of the principal component analysis at the genus level; (**c**–**e**) the relative proportions of community abundance at the phylum, genus, and species levels.

The composition analysis of gut microbiota among the blank, FOS, RG-46, RG-56, and RG-67 groups is shown in Figure 3. The Venn diagram (Figure 3a) among different groups was used to count the number of common and unique OTUs in multiple samples, showing the similarities and overlaps of OTUs more intuitively. A total of 258 OTUs were shared by the five groups, and each group had its own unique OTUs. Principal component analysis at the genus level (Figure 3b) reflected a large difference in the microbiota composition of the blank, FOS, and pectic polysaccharide groups. However, RG-46, RG-56, and RG-67 performed similarly in the PCA analysis, which may be due to a minor disparity in their structures. 

The relative proportions of community abundance at the phylum, genus, and species levels are shown in Figure 3c–e. Bacteroidota, Firmicutes, Proteobacteria, and Actinobacteria were the main phyla in microbiota, accounting for over 98% [36]. After 24 h of fermentation, the abundances of Bacteroidota and Actinobacteriota increased in the FOS and RG-I pectic polysaccharide groups. As a strong competitor in the gut ecosystem, Bacteroidota is involved in many important metabolic activities in the human colon, including carbohydrate fermentation, nitrogen utilization, and bioconversion of bile acids and other steroids [37,38]. Apparently, RG-I pectic polysaccharides are comparable to traditional prebiotics such as FOS in promoting Bacteroidota growth. However, the four samples inhibited the development of Proteobacteria, including the pathogenic bacteria *Salmonella*, *Escherichia coli*, and *Shigella*. 

According to the result of community abundance at the genus level (Figure 3d), the composition of gut microbiota changed significantly compared with the blank group. FOS and the RG-I pectic polysaccharides markedly enhanced the relative abundances of *Prevotella*, *Bifidobacterium*, and *Lactobacillus* and substantially decreased the relative abundances of *Escherichia-Shigella*, *Klebsiella*, and *Megamonas*. As traditional probiotics, *Bifidobacterium* and *Lactobacillus* proliferated in the three RG-I pectic polysaccharides, and the high-RG-I domain pectic polysaccharides performed better. The dominant bacteria in RG-46, RG-56, and RG-67 were *Bacteroides*, *Prevotella*, *Phascolarctobacterium*, and *Bifidobacterium*, showing a similar microbiota composition to that of the FOS group. All RG-I pectic polysaccharides showed stronger effects in promoting Bacteroides and *Phascolarctobacterium*, indicating that these two bacteria may play a crucial role in the degradation and utilization of pectic polysaccharides. Meanwhile, they led to a reduction in the abundances of *Escherichia-Shigella* and *Klebsiella*, indicating that RG-I pectic polysaccharides possessed conspicuous abilities in hindering the growth and breeding of conditional pathogens.

The community abundance at the species level (Figure 3e) could provide more detailed information about the composition of gut microbiota. A huge discrepancy existed among the blank, FOS, and RG-I pectic polysaccharide groups, although the microbial community structures of RG-46, RG-56, and RG-67 were homogeneous. All RG-I pectic polysaccharides could significantly facilitate the growth of specific bacteria, such as *Bacteroides_vulgatus*, *metagenome_g_Prevotella*, *Phascolarctobacterium_faecium*, *Bifidobacterium_pseudocatenulatum*, and *Bacteroides_thetaiotaomicron*. These bacteria were the main microflora of the degraded pectic polysaccharides, and the high content of the RG-I domain affected their growth and propagation. The most obvious evidence for this is that the abundance of *Bacteroides_vulgatus* and *Bifidobacterium_pseudocatenulatum* in RG-67 and RG-56 was evidently higher than that in RG-46.

### 3.5. Group Variation Analysis of Gut Microbiota

**Figure 4:**
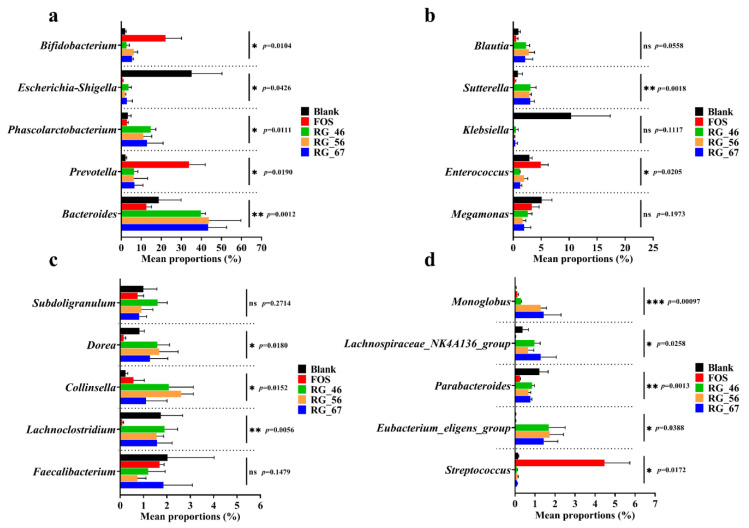

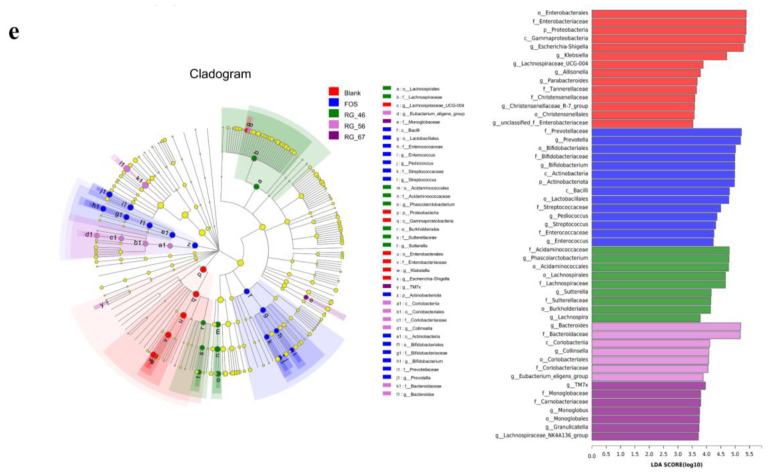
Comparison analysis among five groups of gut microbiota. (**a**–**d**) One-way ANOVA results based on the abundance of the top 20 genera; (**e**) linear discriminant analysis effect size (LEfSe) multilevel discriminant analysis of microbiota differences. Statistically significant differences among different samples are shown with asterisks, as follows: (*) *p* < 0.05, (**) *p* < 0.01, and (***) *p* < 0.001.

Figure 4a–d show the results of the one-way ANOVA with Welch’s post hoc test based on the abundance of the top 20 genera among the five groups. Figure 4a shows the five genera with the highest abundance in the gut microenvironment, indicating that RG-I-type pectic polysaccharides had different abilities in modulating gut microbiota. 

The traditional prebiotic FOS showed outstanding capacities in promoting *Bifidobacterium* and *Prevotella*. As a traditional probiotic, *Bifidobacterium* plays an important role in the biological barrier, antitumor effect, immune function, and gut health [39]. *Prevotella* is a key bacterium in host–microbiome interactions, particularly in relation to nutrition and complex carbohydrate metabolism [40]. 

RG-46, RG-56, and RG-67 also showed the ability to stimulate these bacteria’s growth. Interestingly, *Bacteroides* and *Phascolarctobacterium* showed distinct preferences for RG-I-type pectic polysaccharides. In addition to the above bacteria, all RG-I pectic polysaccharides could selectively promote *Blautia*, *Sutterella*, *Dorea*, *Collinsella*, *Monoglobus*, and *Eubacterium_eligens_group*. Most of the dominant bacteria in RG-I polysaccharides are involved in the degradation of carbohydrates and the generation of SCFAs, benefiting gut health through multiple mechanisms. For example, Blautia has potential probiotic properties, such as preventing inflammation and promoting the production of SCFAs to maintain intestinal homeostasis [41]. 

*Sutterella*, *Dorea*, and *Monoglobus* are related to the fermentation of pectin, consistent with literature reports [42]. Among them, *Monoglobus* pectinilyticus was identified as a pectin-degrading specialist bacterium that participates in degrading various pectins, RG-I, and galactans to produce degradation products that are presumably shared with other bacteria [43]. Another bacterium with specificity for RG-I pectic polysaccharides is *Eubacterium_eligens_group*, which was significantly abundant in RG-46, RG-56, and RG-67 but hardly existed in the FOS and blank groups. We came to the same conclusion as other studies that the growth of *Eubacterium_eligens_group* and *Blautia* occurs only with pectic polysaccharides [44]. Notably, *Eubacterium_eligens_group* is related to butyrate production, but the effects and specific mechanisms in maintaining human health and pectin degradation remain unclear [45,46].

Linear discriminant analysis effect size (LEfSe) multilevel discriminant analysis (Figure 4e) is a useful tool for discovering biomarkers among different groups. Each group had its specific biomarkers, and linear discriminant analysis (LDA) scores of those biomarkers were calculated. Only biomarkers with LDA scores greater than 3.5 are shown in Figure 4. *Phascolarctobacterium*, *Sutterella*, and *Lachnospira* were the main biomarkers in RG-46 at the genus level. RG-56 and RG-67 also possessed their typical genera as biomarkers. In RG-56, they were *Bacteroides*, *Collinsella* (butyrate producer), and *Eubacterium_eligens_group* [47]. However, in RG-67, the biomarkers were *TM7x*, *Monoglobus*, *Granulicatella*, and *Lachnospiraceae_NK4A136_group*. This finding indicates that the proportion of the RG-I domain and high neutral sugar contents affected the modulation of intestinal microbial communities, as shown by the changes in the dominant bacteria and biomarkers.

### 3.6. Function Prediction and Pearson Correlation Analysis

**Figure 5:**
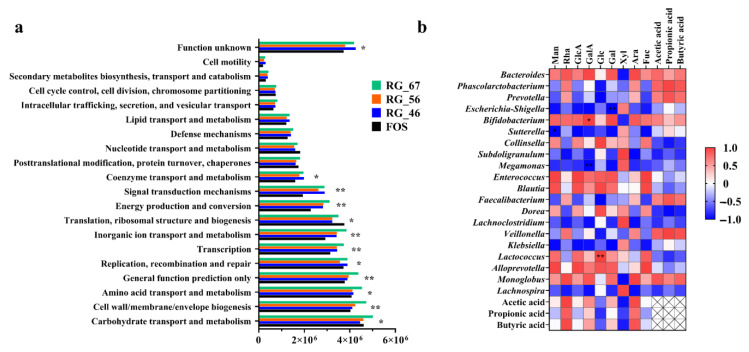
Results of PICRUSt function prediction analysis and Pearson correlation analysis. (**a**) Clusters of orthologous groups (COG) function annotation analysis; (**b**) the heatmap result based on Pearson’s correlation coefficient among monosaccharides, SCFAs, and the relative abundances of bacteria at the genus level. Statistically significant differences are shown with asterisks, as follows: (*) *p* < 0.05 and (**) *p* < 0.01.

PICRUSt was used to standardize the OTU abundance table, and then the corresponding green gene ID of each OTU was used to annotate its clusters of orthologous groups (COG) functions. This method can offer annotation information on the function levels and their abundance in different groups. FOS served as the control group, and the functional abundances of RG-46, RG-56, and RG-67 are compared in Figure 5a. The abundance of the COG function in RG-67 was higher than in FOS and the other RG-I pectic polysaccharides, especially in carbohydrate transport and metabolism, cell wall/membrane biogenesis, and energy production and conversion. Combined with its abilities in SCFA production and microbiota modulation, RG-67 may have the best performance in promoting energy metabolism and maintaining intestinal health.

The correlation analysis (Figure 5b) of the intestinal microflora composition, monosaccharides, and SCFAs showed that monosaccharides played a crucial role in regulating bacteria and generating SCFAs. For the high-RG-I domain pectic polysaccharides, Ara, GalA, Gal, and Rha were the core components, conferring polysaccharides special functions and promoting the growth of probiotics. GalA, as the monosaccharide with the highest proportion among the polysaccharides, maintained a positive relationship with *Bifidobacterium* (*p* < 0.05) and a negative relationship with *Megamonas* (*p* < 0.01). The presence of Ara was highly positively correlated with the development of *Bacteroides*, *Bifidobacterium*, and *Monoglobus*, and was also connected with the accumulation of acetic acid, propionic acid, and butyric acid. Another neutral sugar, Gal, also had unique effects in affecting gut microbiota, such as restraining the reproduction of *Escherichia-Shigella* (*p* < 0.01), *Subdoligranulum*, *Megamonas*, *Lachnoclostridium*, *Klebsiella*, and *Lachnospira*. In the results based on RG-46, RG-56, and RG-67, it was found that Rha, Ara, GalA, and Gal had better capacities than the other monosaccharides in this aspect. Ara and Rha were fundamental to the RG-I domain, and their contents determined the polysaccharides’ functional characteristics and the regulation of gut microbiota to some extent. The above might explain why RG-67 performed better in SCFA generation and modulation of gut microbiota. Furthermore, *Bacteroides*, *Phascolarctobacterium*, *Prevotella*, *Bifidobacterium*, and *Monoglobus* were positively correlated with the formation of SCFAs.

## 4. Conclusions

Three different pectic polysaccharides (RG-46, RG-56, and RG-67) with high RG-I domain proportions recovered from citrus canning processing wastewater were used in this study, and the relationship between the RG-I domain and in vitro fermentation characteristics was investigated. Structurally, the main difference among RG-46, RG-56, and RG-67 was the proportion of the RG-I domain. The fermentation results showed that the RG-I domain was significantly related to pectic polysaccharides’ fermentation characteristics, especially in SCFA generation and modulation of gut microbiota. RG-67 performed better than RG-56 and RG-46 in producing acetate, propionate, and butyrate. The main bacteria that participated in degrading the RG-I pectic polysaccharides were *Bacteroides*, *Phascolarctobacterium*, *Bifidobacterium*, and *Blautia*. Furthermore, *Eubacterium_eligens_group* and *Monoglobus* showed particular preferences for RG-I-type pectic polysaccharides, and their abundances were closely related to the proportion of the RG-I domain. Our study had limitations. For example, the structure–function relationship between the RG-I domain of pectic polysaccharides and its effect on health needs to be further determined in vivo. Nevertheless, the present study highlighted the prebiotic potential of RG-I pectic polysaccharides and revealed their salutary influence on human intestinal ecology in vitro. Our finding provide more information about the structure–function relationship and offer feasible ideas for citrus or other food processing enterprises to recover functional ingredients from processing byproducts, which is helpful for the realization of green production and value addition.

## Figures and Tables

**Table 1:** Analysis of monosaccharide composition, molecular weight, and degree of esterification.

		**RG-46**	**RG-56**	**RG-67**
**Monosaccharide composition (mol%)**	**Fuc**	1.18 ± 0.31 ^A^	1.19 ± 0.34 ^A^	1.06 ± 0.18 ^A^
	**Rha**	4.82 ± 0.56 ^A^	4.92 ± 0.76 ^A^	6.27 ± 1.08 ^A^
	**Ara**	26.69 ± 1.89 ^B^	30.57 ± 2.25 ^B^	35.49 ± 0.02 ^A^
	**Gal**	10.27 ± 0.80 ^B^	15.92 ± 2.82 ^A^	19.44 ± 0.86 ^A^
	**Glc**	3.17 ± 0.18 ^A^	2.07 ± 0.16 ^B^	2.37 ± 0.24 ^B^
	**Man**	1.06 ± 0.15 ^C^	1.81 ± 0.38 ^BC^	2.47 ± 0.64 ^A^
	**Xyl**	2.05 ± 0.08 ^A^	0.98 ± 0.17 ^B^	0.80 ± 0.06 ^B^
	**GalA**	50.19 ± 3.45 ^A^	39.68 ± 1.84 ^B^	31.17 ± 1.45 ^C^
	**GlcA**	0.54 ± 0.24 ^B^	2.85 ± 0.17 ^A^	0.94 ± 0.01 ^B^
**HG domain%**		45.36 ± 4.01 ^A^	34.76 ± 4.09 ^B^	24.90 ± 1.34 ^C^
**RG-I domain%**		46.61 ± 3.82 ^C^	56.34 ± 3.34 ^B^	67.46 ± 2.65 ^A^
**Mw (kDa)**		647.4 (±0.4%)	722.0 (±0.8%)	735.9 (±0.4%)
**Mn (kDa)**		227.1 (±0.4%)	675.9 (±8.1%)	757.5 (±4.0%)
**Mw/Mn**		4.3 (±2.9%)	10.7 (±8.2%)	9.7 (±4.0%)
**DM%**		7.11 ± 0.99 ^B^	7.60 ± 1.73 ^B^	11.4 ± 1.17 ^A^
**DA%**		0.28 ± 0.01 ^A^	-	-

The molar percentages (mol%) of homogalacturonan (HG) and rhamnogalacturonan-I (RG-I) were calculated using the following formulas: HG (%) = GalA(mol%) − Rha (mol%); RG-I (%) ≈ 2Rha(mol%) + Ara(mol%) + Gal(mol%). Different uppercase letters indicate significant differences (*p* < 0.05) in the same ingredient among different groups.

**Table 2:** Analysis of alpha diversity in gut microbiota.

		Blank	FOS	RG-46	RG-56	RG-67
**Community richness**	**Sobs**	321.50 ± 0.50 ^A^	274.67 ± 18.93 ^C^	344.67 ± 9.74 ^A^	323.00 ± 23.34 ^A^	321.00 ± 1.63 ^BC^
	**ACE**	390.13 ± 3.25 ^BC^	345.29 ± 28.50 ^C^	421.90 ± 14.33 ^A^	386.07 ± 24.99 ^BC^	393.81 ± 3.96 ^BC^
	**Chao**	406.92 ± 0.14 ^A^	350.89 ± 18.24 ^B^	435.72 ± 16.20 ^A^	385.15 ± 24.92 ^BC^	396.86 ± 13.46 ^BC^
**Community diversity**	**Shannon**	2.83 ± 0.09 ^B^	2.92 ± 0.03 ^B^	3.79 ± 0.09 ^A^	3.67 ± 0.14 ^A^	3.65 ± 0.14 ^A^
	**Simpson**	0.20 ± 0.02 ^A^	0.16 ± 0.01 ^B^	0.05 ± 0.01 ^C^	0.07 ± 0.01 ^C^	0.07 ± 0.01 ^C^
**Community evenness**	**Heip**	0.05 ± 0.00 ^B^	0.06 ± 0.00 ^B^	0.13 ± 0.01 ^A^	0.12 ± 0.01 ^A^	0.12 ± 0.01 ^A^
	**Shannoneven**	0.49 ± 0.01 ^B^	0.52 ± 0.00 ^B^	0.65 ± 0.02 ^A^	0.64 ± 0.02 ^A^	0.63 ± 0.01 ^A^
	**Simpsoneven**	0.02 ± 0.00 ^B^	0.02 ± 0.00 ^B^	0.05 ± 0.01 ^A^	0.05 ± 0.01 ^A^	0.05 ± 0.01 ^A^
**Community coverage**	**Coverage**	1.00 ± 0.00 ^A^	1.00 ± 0.00 ^A^	1.00 ± 0.00 ^A^	1.00 ± 0.00 ^A^	1.00 ± 0.00 ^A^

Sobs represents the observed richness. ACE represents the ACE estimator. Chao represents the Chao1 estimator. Shannon represents the Shannon diversity index. Simpson represents the Simpson diversity index. Heip represents Heip’s metric of community evenness. Shannoneven represents the Shannon index-based measure of evenness. Simpsoneven represents the Simpson index-based measure of evenness. Coverage represents Good’s coverage. Different uppercase letters indicate significant differences (*p* < 0.05) in the same ingredient among different groups.

## Data Availability

The data presented in this study are available on request from the corresponding author.
